# Inducible localized delivery of an anti-PD-1 scFv enhances anti-tumor activity of ROR1 CAR-T cells in TNBC

**DOI:** 10.1186/s13058-022-01531-1

**Published:** 2022-06-03

**Authors:** Micaela Harrasser, Satyen Harish Gohil, Hiu Lau, Marco Della Peruta, Vincent Muczynski, Dominic Patel, Elena Miranda, Kristiana Grigoriadis, Anita Grigoriadis, David Granger, Rachel Evans, Amit Chunilal Nathwani

**Affiliations:** 1 Department of Academic Haematology, University College London Cancer Institute, London, WC1E 6DD UK; 2 Katharine Dormandy Haemophilia and Thrombosis Centre, Royal Free NHS Trust Pond Street, London, NW3 2QG UK; 3 Biobank and Pathology Translational Technology Platform, CRUK-UCL Centre, Cancer Institute, University College London, London, WC1E 6DE UK; 4 Breast Cancer Now Research Unit, King’s College London, Guy’s Hospital, Great Maze Pond, London, SE1 9RT UK; 5 NovalGen Ltd, University College London, London, NW3 2QG UK; 6 Comprehensive Cancer Centre, King’s College London, London, SE1 1UL UK

**Keywords:** ROR1, CAR-T cell therapy, PD-1, Checkpoint blockade, F i-CAR-T cell, Single-chain variable fragment (scFv), TNBC, Solid tumors, Inducible secretion

## Abstract

**Background:**

Chimeric antigen receptor (CAR)-T cells can induce powerful immune responses in patients with hematological malignancies but have had limited success against solid tumors. This is in part due to the immunosuppressive tumor microenvironment (TME) which limits the activity of tumor-infiltrating lymphocytes (TILs) including CAR-T cells. We have developed a next-generation armored CAR (F i-CAR) targeting receptor tyrosine kinase-like orphan receptor 1 (ROR1), which is expressed at high levels in a range of aggressive tumors including poorly prognostic triple-negative breast cancer (TNBC). The F i-CAR-T is designed to release an anti-PD-1 checkpoint inhibitor upon CAR-T cell activation within the TME, facilitating activation of CAR-T cells and TILs while limiting toxicity.

**Methods:**

To bolster potency, we developed a F i-CAR construct capable of IL-2-mediated, NFAT-induced secretion of anti-PD-1 single-chain variable fragments (scFv) within the tumor microenvironment, following ROR1-mediated activation. Cytotoxic responses against TNBC cell lines as well as levels and binding functionality of released payload were analyzed in vitro by ELISA and flow cytometry. In vivo assessment of potency of F i-CAR-T cells was performed in a TNBC NSG mouse model.

**Results:**

F i-CAR-T cells released measurable levels of anti-PD-1 payload with 5 h of binding to ROR1 on tumor and enhanced the cytotoxic effects at challenging 1:10 E:T ratios. Treatment of established PDL1 + TNBC xenograft model with F i-CAR-T cells resulted in significant abrogation in tumor growth and improved survival of mice (71 days), compared to non-armored CAR cells targeting ROR1 (F CAR-T) alone (49 days) or in combination with systemically administered anti-PD-1 antibody (57 days). Crucially, a threefold increase in tumor-infiltrating T cells was observed with F i-CAR-T cells and was associated with increased expression of genes related to cytotoxicity, migration and proliferation.

**Conclusions:**

Our next-generation of ROR1-targeting inducible armored CAR platform enables the release of an immune stimulating payload only in the presence of target tumor cells, enhancing the therapeutic activity of the CAR-T cells. This technology provided a significant survival advantage in TNBC xenograft models. This coupled with its potential safety attributes merits further clinical evaluation of this approach in TNBC patients.

**Supplementary Information:**

The online version contains supplementary material available at 10.1186/s13058-022-01531-1.

## Background

Chimeric antigen receptor (CAR) T cells comprise autologous T cells genetically engineered to express a synthetic receptor to enable HLA-independent recognition of target antigens, resulting in T cell activation and target cell cytotoxicity. CAR-T cells directed against the B cell antigen CD19 demonstrate dramatic clinical efficacy against relapsed and refractory B cell malignancies and have become an established therapeutic option for these patients [1].

In contrast, targeting of solid tumors with CAR-T cells has shown limited or transient efficacy, especially for those with high unmet need including triple-negative breast cancer (TNBC) [2]. Compared to other breast cancer subtypes, effective TNBC treatment has been hampered by a lack of targetable antigens and aggressive growth, resulting in a high mortality rate. We focused on receptor tyrosine kinase-like orphan receptor 1 (ROR1), a cell surface antigen expressed on a range of malignancies, including TNBC, and associated with aggressive disease, poor prognosis and stem cell features that may contribute to chemotherapeutic resistance and relapse [3].

A further obstacle in effective treatment of TNBC is the immunosuppressive solid tumor microenvironment (TME), which induces sustained upregulation of inhibitory checkpoints such as PD-1/PD-L1 on tumor-infiltrating lymphocytes (TILs). Antibody-mediated checkpoint blockade (CPB) of the PD-1/PD-L1 axis has been a major breakthrough in the treatment of many cancers, including TNBC [4]. There is accumulating evidence that like TILs, CAR-T cells also acquire a terminally differentiated and exhausted phenotype associated with increased expression of multiple inhibitory checkpoints including PD-1 [5]. As such, there is a great deal of interest in combining the use of CPB to modulate the immunosuppressive TME and bolster the potency of CAR-T cells and initial studies are encouraging [6] but associated with significant systemic toxicity.

Recently, CD19- and MUC16-specific CAR-T cells with constitutive secretion of anti-PD-1 scFvs showed augmented functionality and enhanced killing of syngeneic murine tumor targets compared to CAR-T cells combined with systemic anti-PD-1 antibody [7]. Encouraged by these results we developed a prototypic ROR1-targeting CAR construct in which expression of a human anti-PD-1 scFv was under the control of a nuclear factor of activated T cell (NFAT)-sensitive promoter (F i-CAR-T cells). This approach aims to ensure that secretion of anti-PD-1 scFvs from transgenic T cells only occurs in the TME following engagement with ROR1 on tumor cells. This inducible characteristic of F i-CAR-T cells would restrict PD-1 signaling blockade to the site of action, differentiating it from previously described approaches [7] in which armored CARs constitutively secreted anti-PD-1 scFvs, raising the potential for systemic PD-1 antagonism. We demonstrate that F i-CAR-T cells were capable of more potent killing of ROR1-positive TNBC-like tumors than ROR1 CAR-T cells used in combination with anti-PD-1 monoclonal antibodies. These data are consistent with the use of our CAR-T cells to deliver immune modulatory scFvs within the TME with the safety of avoiding systemic blockade.

## Results

### Inducible blockade of PD-1/PD-L1 pathway to enhance potency of F CAR-T cells

**Fig. 1:**
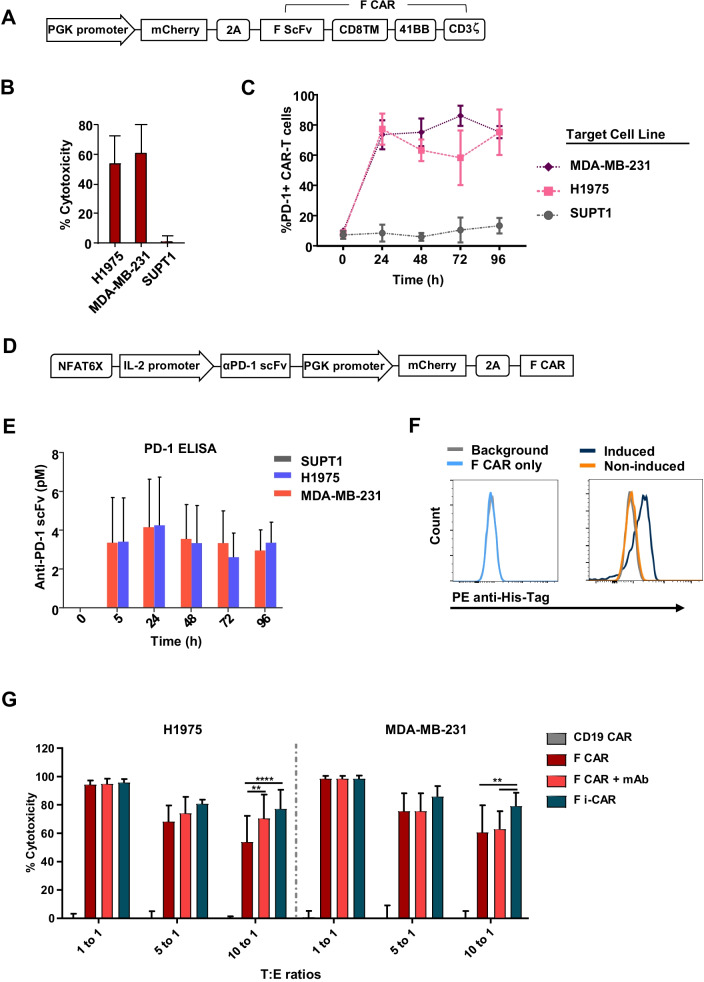
Regulated PD-1 blockade in ROR1 CAR-T cells—an inducible system. **A** Transgene schematic of ROR1-targeted F CAR lentiviral construct. **B** F CAR-mediated killing was assessed at 72 h at 10:1 target/effector (T/E) ratio, mean + SD of 3 donor-derived CAR-T cells. **C** PD-1 expression of F CAR-T cells assessed via flow cytometry at 0 h (without target cells) and every 24 h during co-culture with the cell lines MDA-MB-231 and H1975 (both ROR1 +) or the ROR1-negative cell line SUPT1 at a 10:1 T/E ratio. Results show mean + SD of 3 donor-derived CAR-T cells. **D** Transgene schematic of the F i-CAR lentiviral construct. **E** Supernatant from co-cultures was collected and secreted anti-PD-1 scFv quantified via PD-1 ELISA. Results show mean ± SD of 3 donors in triplicates. **F** Flow cytometry binding assay of secreted aPD-1 scFv to SupT1-PD-1 + cells shows specific binding represented by PE-anti-His Tag shift (right plot, blue histogram) compared to background, negative control (supernatant from F CAR co-culture, light blue histogram) and non-induced secretion (F i-CAR with ROR1 cell co-culture, orange histogram). **G** Long-term 72 h co-cultures of F CAR-T cells, F i-CAR-T cells or F CAR-T cells CAR + 10 µg/mL anti-human PD-1 antibody (F CAR + mAb), against MDA-MB-231 and H1975 cell lines, mean ± SD of 3 donors normalized to negative control CD19 CAR; two-way ANOVA used: *p < 0.05; **p < 0.005; ***p < 0.0005; ****p < 0.0001

We cloned a humanized ROR1 scFv into a bicistronic second-generation CAR backbone containing 41BB-CD3ζ intracellular signaling domains and mCherry transduction marker (F CAR; Fig. 1A). F CAR-T cells co-cultured with ROR1 + cell lines (MDA-MB-231 and H1975) demonstrated efficient targeted cytotoxicity 72 h post-co-culture (Fig. 1B), which was associated with a rapid, sevenfold to ninefold upregulation of surface PD-1 within 24 h correlating with T cell activation, compared with ROR1-negative cells (SUPT1) or stimulation with exogenous IL-2 alone. PD-1 expression remained elevated throughout the course of the co-culture (Fig. 1C).

We hypothesized that localized blockade of PD-1/PD-L1 axis would improve F CAR-T cell efficacy. We first cloned the variable regions of the PD-1-blocking antibody, pembrolizumab, in an scFv format into a bicistronic expression cassette (Additional file 1: Figure SI 1A). We confirmed secretion of the PD-1 scFv from transfected HEK-293 T cells using flow cytometry (Additional file 1: Figure SI 1B) and confirmed high affinity binding by surface plasmon resonance (ka(1/Ms) = 6.26 ± 0.77 × 10^5^, kd(1/s) = 2.26 ± 0.2 × 10^–3^, K_D_(M) = 3.62 ± 0.215 × 10^–9^, Additional file 1: Figure SI 1C).

To make secretion of the anti-PD-1 scFv dependent on CAR-T cell activation, we cloned the scFv downstream of 6 nuclear factor of activated T cell (NFAT) binding sites, followed by the minimal IL-2 promoter (F i-CAR; Fig. 1D). Gene transfer efficiency of primary human T cells with the F i-CAR construct was comparable to the parental F CAR expression cassette (Additional file 1: Figure SI 2A for mCherry expression). Co-culture of F i-CAR-T cells with ROR1 + PD-L1 + tumor cell lines (Additional file 1: Figure SI 2B) demonstrated average production of 4 pM of anti-PD-1 scFv, with expression maintained over time as assessed by ELISA (Fig. 1E). In parallel, we confirmed supernatant from co-culture experiments contained sufficient scFv to be detectable by flow cytometry (Fig. 1F). Finally, we generated a Gaussia luciferase-tagged anti-PD-1 scFv and observed similar kinetics of anti-PD-1 scFv secretion (Additional file 1: Figure SI 2C). Importantly, anti-PD-1 scFv was undetectable in co-cultures with ROR1-negative SUPT1 demonstrating specificity.

### Induced secretion of anti-PD-1 scFv by ROR1 CAR-T cells enhances targeted cytotoxicity

We assessed efficacy of F i-CAR-T cells compared to conventional F CAR-T cells and, as a control, F CAR-T cells combined with anti-PD-1 antibody. At a 10:1 T/E ratio, F i-CAR-T cells were more efficient than conventional F CAR (79% vs 60% for MDA-MB-231, *p* = 0.013 and 78% vs 56% for H1975 cells, *p* < 0.001, Fig. 1G). Combination of F CAR and full-length antibody showed higher activity than F CAR alone, but this was still lower than the efficacy of F i-CAR-T cells (Fig. 1G). This suggests that inducible secretion of anti-PD-1 scFv significantly enhances cytotoxicity at lower and more physiologically relevant T/E ratios. We saw no increase in off-target cytotoxicity against ROR1-negative SUPT1 and MCF-7 cell lines compared to control CD19 CAR-T cells (Additional file 1: Figure SI 2D). In keeping with autocrine localized secretion of a blocking PD-1 scFv, PD-1 expression (relative to CD8 + cells or total) on F i-CAR-T cells was twofold lower compared to F CAR-T cells (5% vs 18.6% for expression on CD8 + cells, *p* = 0.02; and 12% vs 27% for total expression, *p* = 0.0065), while combination therapy showed higher PD-1 expression than F i-CAR-T cells (7.8% on CD8 + cells and 16% total PD-1). Other inhibitory checkpoint receptors TIM-3, LAG-3 and CTLA-4 showed similar levels of expression (Additional file 1: Figure SI 2E). Overall, these experiments demonstrate improved in vitro cytotoxicity following disruption of the PD-1/PD-L1 axis.

### Treatment of TNBC xenograft mice with ROR1 i-CAR-T cells reduces tumor growth and increases survival

To assess efficacy of F CAR-T cells in vivo, we used the TNBC MDA-MB-231 xenograft model, engineered to express firefly luciferase. Tumor engraftment was confirmed with bioluminescence imaging 24 h prior to treatment with F CAR-T cells, F CAR-T cells + systemic anti-PD-1 antibody or F i-CAR-T cells alone. A control cohort of animals received CD19 i-CAR-T cells designed to secrete the same anti-PD-1 scFv following antigen binding. All treatment protocols were well tolerated, and tumor burden was monitored over the duration of the experiment with no evidence of off-tumor, on-target toxicity (Additional file 1: Figure SI 3C).

**Fig. 2:**
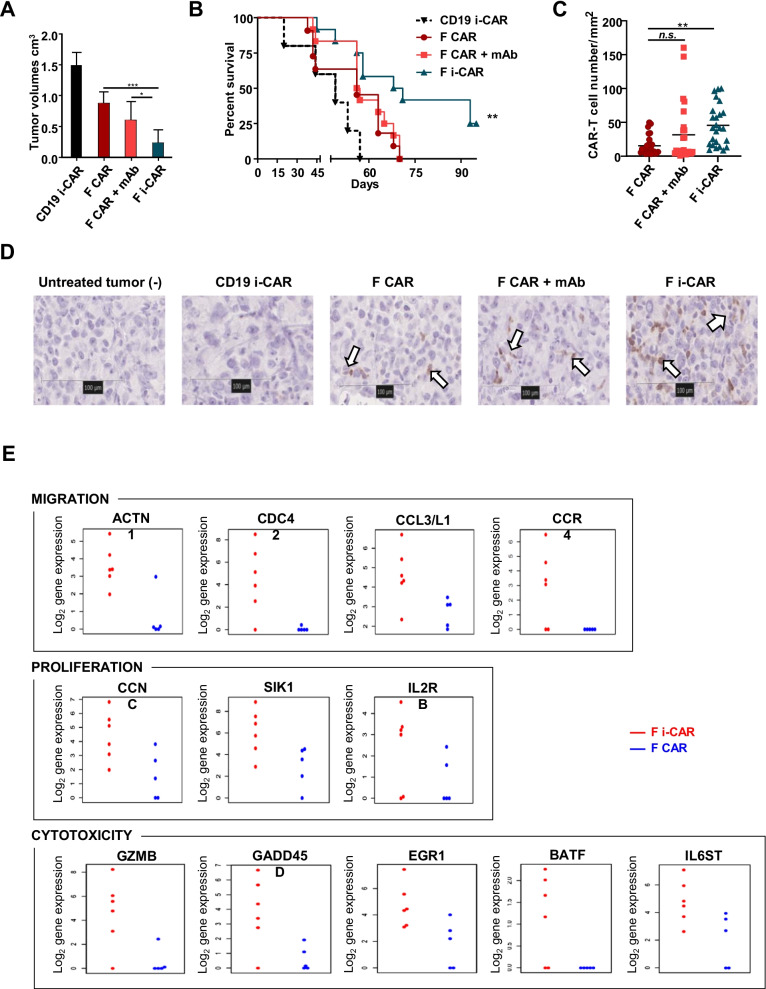
Treatment of TNBC xenograft mice with F i-CAR-T cells reduces tumor growth and increases survival. 2 × 10^6^ MDA-MB-231-ffLuc + cells were injected in the mammary fat pad of NSG mice. 6 days later, BLI was performed to ensure uniform engraftment of tumor cells. On day 7, 4 × 10^6^ CAR-T cells were injected intravenously with or without 250 µg/mouse anti-PD-1 mAb intraperitoneally on day 7, 10 and 14. Tumors were measured with BLI (up to week 4 post-treatment) and digital caliper once tumors were palpable. **A** Tumor volumes (caliper measurement) at 4 weeks post-treatment, 6 representative mice/group displayed with two-way ANOVA performed *p = 0.04, ***p = 0.0005; **B** Kaplan–Meier survival curve of mice (*n* = 5–12 per group) treated with F CAR, F CAR + mAb, F i-CAR or CD19 i-CAR; log-rank test used: **p = 0.001; **C** CAR-T cell numbers per mm^2^, total of 5 sections/tumor, 5 tumors/treatment group(*n* = 25); one-way ANOVA used with F CAR as comparator arm, **p < 0.01; **D** tumors were harvested and processed for immunohistochemistry: representative mCherry staining on tumors from each treatment group (40 ×); **E** tumor-bearing mice were killed 4 weeks post-CAR-T cell treatment, with TILs extracted and sorted based on mCherry expression for downstream analysis: genes involved in migration (top panel), proliferation (middle panel) and cytotoxicity (bottom panel) were selected according to Log_2_ fold-change and *p* < 0.05. Differentially expressed genes from each tumor TIL population depicted as single data points and shown as Log_2_ gene expression

Four weeks post-inoculation, mice treated with F CAR-T cells alone or F CAR-T cells combined with anti-PD-1 antibody had reduced mean tumor burden of 0.88 ± 0.2 cm^3^ and 0.6 ± 0.3 cm^3^, respectively. This was approximately twofold lower than the mean tumor burden in the control cohorts treated with CD19 i-CAR and was confirmed by BLI imaging. In contrast, mice treated with F i-CAR-T cells showed a significantly reduced mean tumor burden of 0.26 ± 0.21cm^3^ (Fig. 2A; Additional file 1: SI 3A-B). This was associated with significantly superior survival, increased from a median of 44 days in CD19 i-CAR-T cell treatment group, to 49 and 57 days in F CAR-T cell and F CAR-T cell + anti-PD-1 antibody treatment groups, respectively. A further increase to 71 days was demonstrated in mice treated with F i-CAR-T cells (Fig. 2B).

### Ex vivo analysis reveals that F i-CAR-T cells demonstrate improved tumor penetration and display a unique transcriptional signature

To better understand the mechanisms for slower tumor growth and increased survival, tumor-infiltrating T cells were profiled from MDA-MB-231 tumors treated with CAR-T cells. CAR-T cells were detected by immunohistochemical detection of mCherry (Fig. 2D) which confirmed a threefold increase in F i-CAR-T cells within the TNBC xenografts (Fig. 2C) associated with an increase in non-CAR CD8+ T cells (Additional file 1: Figure SI 3D).

We next performed gene expression profiling of F and F i-CAR-T cells isolated from MDA-MB-231 tumors. Several genes involved in migration (*ACTN1, CDC42, CCL3/L1, CCR4*), proliferation (*CCNC, SIK1, IL2RB*) and cytotoxicity (*GZMB, GADD45D, EGR1, BATF, IL6ST*) were significantly increased in F i-CAR-T cells compared to F CAR-T cells (Fig. 2E). Additionally, F i-CAR-T cells were enriched in cytotoxic T cells (4.5 vs 4.2) and reduced in T-regulatory cells (3.9 vs 4.6) and exhausted CD8 T cells (3.9 vs 4.1, all log_2_ raw count, Additional file 1: Figure SI 4A). Finally, gene pathway scoring analysis showed increased cell cycle and antigen presentation, and decreased apoptosis, autophagy and exhaustion for infiltrating F i-CAR-T cells (Additional file 1: Figure SI 4B). Overall, the profiling of tumor-infiltrating CAR-T cells suggests that improved anti-tumor effect of the F i-CAR-T cells in MDA-MB-231 tumors may be due to increased T cell infiltration, proliferation and a more inflammatory phenotype enabling more robust targeting of ROR1 + tumor cells.

## Materials and methods

### Lentiviral constructs

The humanized scFv against human ROR1 has been previously described [8], and we used the fmc63 anti-CD19 scFv as the control. The CAR constructs comprise the respective scFv linked to the hinge region of human IgG1, CD8α transmembrane sequence and 41BB and CD3ζ intracellular co-stimulatory domains [9]. The sequence of the anti-PD-1 antibody pembrolizumab was obtained from the IMGT website (IMGT entry ID: 5jxe) and engineered into a scFv format, consisting of the variable heavy and variable light chains linked with a short amino-acid linker sequence (www.imgt.org). To aid purification and detection, we included a C-terminal hexa-histidine (His)-tag. The inducible expression cassette followed the protocol detailed by Hooijberg and colleagues [10], comprising 6 nuclear factors of activated T cell (NFAT)-binding sites, the minimal IL-2 promoter followed by the anti-PD-1 scFv. The lentiviral backbone for the F i-CAR was purchased from Addgene (#17616), and firefly and Gaussia luciferase sequences were cloned from commercial vectors (Promega, #E1310, and ThermoScientific, #16146).

### Lentiviral production

Functional lentiviral particles are generated by co-transfecting confluent human embryonic kidney (HEK) 293-T cells with a second-generation lentiviral packaging system (pMD2.G for the envelope and pCMV delta R8.2 for the packaging, both from Addgene) and the desired transfer plasmid with GeneJuice following the manufacturer’s instructions (Merck). Supernatant was harvested after 48 h and used immediately or stored at −80 °C.

### Fresh T cell isolation and transduction

Peripheral blood mononuclear cells were obtained after centrifugation of fresh blood, obtained from healthy volunteers, on a density gradient using Ficoll-Paque Plus (GE Healthcare). Isolated PBMCs were activated with CD3/CD28 Dynabeads (ThermoScientific, #11141D) with IL-2 (Miltenyi), and transduction was performed on RetroNectin-coated plates (Takara, #T100A/B).

### Cell lines and reagents

HEK-293 T and SUPT1 cells were obtained from American Type Culture Collection (ATCC; LGC Standards). MCF-7 cell line was obtained from Deutsche Sammlung von Mikroorganismen und Zellkulturen (DSMZ). MDA-MB-231 were from a master cell bank within our institute and was validated by ATCC. H1975 were kindly gifted by Professor Tony Ng (King’s College London, UK). HEK-293 T cells were maintained in Iscove's Modified Dulbecco's Medium (ThermoScientific) supplemented with 10% fetal bovine serum (FBS) (ThermoScientific). All other cell lines and primary cells were maintained in Roswell Park Memorial Institute (RPMI)-1640 medium supplemented with 10% FBS, GlutaMAX and 25 mM HEPES (ThermoScientific). Cells were thawed from master stocks and used for up to 10 passages and maintained at 37 °C with 5% CO2. Cell lines were screened for mycoplasma to ensure negativity.

### Protein purification

His-tagged scFv production was carried out by stably transduced HEK-293 T cells with lentiviral particles in the presence of polybrene (Merck). Culture media was changed 24 h post-transduction to production media (Dulbecco’s Modified Eagle’s Medium + 1% Insulin–Transferrin–Selenium, ThermoScientific), which was harvested every day for up to 4 days. Supernatant was centrifuged and filtered (0.4 µm) to remove cell debris. scFv-His was purified by fast protein liquid chromatography (FPLC) using Excel Ni 5 ml columns with an AKTA Explorer (GE Healthcare Life Sciences). The quality of purification was assessed by Western blot after SDS-PAGE using an HRP-conjugated anti-His antibody (Biolegend, 652504); quantification was performed via Western blot using a purified His-tagged protein. ImageJ software was used for data analysis. Binding of the purified scFv was assessed via flow cytometry using a PE-conjugated anti-His antibody (Bio-techne, IC050P).

### Surface Plasmon resonance

To assess anti-PD-1 scFv binding affinity, we used the Biacore X100 SPR System (GE Healthcare). Briefly, HEK-293 T cells were transiently transfected to produce the anti-PD-1 scFv tagged with a murine Fc stalk. We coupled this scFv-Fc to a CM5 chip with immobilized anti-murine Fc antibody using the mouse antibody capture kit (GE Healthcare). We calculated binding affinities by adding increasing concentrations of recombinant PD-1 (Acro Bioscience) in a single-step multicycle protocol. To generate a baseline/control, we used a chip containing an immobilized antihuman Fc to which the same reagents and protocols were applied. Three independent runs with two fresh scFv-Fc supernatants were performed. Data were analyzed with X100 software (GE).

### Production quantification via ELISA

To quantify the secreted scFv, an in-house PD-1-based ELISA was developed. The standard curve was generated using serial dilutions of FPLC-purified scFv. PD-1 protein (R&D Biosystems, 1086-PD-050) was coated at 1 µg/mL of carbonate buffer overnight at 4 °C. All subsequent incubation steps were incubated at 37 °C. We used TBS + 0.1% Tween-20 for the washing steps. Blocking was performed using TBS-Tween + 3% filtered BSA (Sigma) for 1 h. Samples were incubated for 4 h, followed by incubation with anti-His HRP-conjugated antibody (1:10,000 dilution). Revelation was performed using OPD (Sigma) for 5–10 min prior stopping the reaction with sulfuric acid. Absorbance was read at 492 nm via plate reader (SpectraMax® i3).

### Flow cytometry and antibodies

Data were captured on an LSR Fortessa II flow cytometer (Becton Dickinson) and analyzed using FlowJo software (FlowJo LLC). Cell sorting was undertaken on a FACSAria Cell Sorter (Becton Dickinson). The following antibodies and respective isotype controls were purchased from Biolegend: APC/PE antihuman ROR1 antibody (2A2), Pacific Blue antihuman CD45 (HI30), PercP/Cy5.5 antihuman CD4 (OKT4), PE antihuman CD3 (HIT3a), FITC/PE antihuman CD8 (SK1), Ultra-LEAF Purified or FITC antihuman CD279 (PD-1, clone EH12.2H7), LEAF Purified/PE antihuman CD274 (PD-L1, 29E.2A3). Alexa Fluor® 647 AffiniPure Goat Anti-Human IgG, F(ab')2 fragment specific (109-605-006) was purchased from Jackson Immunolabs and Fixable Viability Dye eFluor® 780 was from ThermoScientific (65-0865).

### Co-culture assays

Short-term co-culture assays were performed in 96-well plates, containing 1 × 10^4^ adherent target cells and CAR-T cell numbers based on the specified target-to-effector (T/E) ratio. Long-term co-cultures were set up using 24-well plates with 4 × 10^4^ adherent target cells. To assess cytotoxicity, we performed a flow cytometry-based killing assay: Briefly, plates were washed, and target cells were harvested and transferred into tubes (microRack II system, Greiner Bio-One Ltd.) containing counting beads (Beckman Coulter, 6605359), viability dye and additional antibodies specified in figure legends. Events were recorded based on bead counts which were used to normalize the numbers of viable cells. Control wells with nonspecific CD19 CAR-T cells were used as the 100% viable cell comparison.

### Immunohistochemistry

Tumor from mice were harvested and placed into 10% neutral buffered formalin (Cellpath)**.** mCherry staining and quantification were performed by UCL Pathology. In brief, deparaffinized hydrated tissue sections underwent antigen unmasking in Tris–EDTA pH 9 (Dako) at high pressure in a pressure cooker for 8 min. After washing and quenching, sections were blocked in 2.5% Horse Serum (Vector ImmPRESS Kit) for 20 min at room temperature. Incubation with primary antibody anti-mCherry (Abcam, ab167453, 1 µg/ml) was for 60 min at room temperature, secondary antibody (anti-Rabbit IgG Polymer Detection Kit, MP-7401, Vector Laboratories) for 30 min at room temperature and DAB + substrate/chromagen (Dako) for 5 min at room temperature prior to counterstaining and mounting.

Slides were scanned in the Hamamatsu NanoZoomer S210 Digital slide scanner. The image analysis has been performed on the whole section with the positive cell counting algorithm from QuPath image analysis software.

Fixation, embedding and CD8 staining were performed at the UCL Institute of Neurology, using the Ventana Discovery XT instrument and Ventana DAB Map detection Kit (760-124). For pre-treatment, Ventana CC1 (950-124) was used. The CD8 antibody (Dako M7103) was used at 1:100 dilution for 1 h before Rabbit anti-murine secondary antibody (Dako E0354) was used.

### Statistics

Statistical analysis was undertaken as specified in the figure legends using GraphPad Prism Version 7–8 for Windows. Statistical significance was taken when *p* < 0.05. At least two independent experiments with different donor T cells were undertaken for all in vitro experiments. For in vivo experiments, at least 2 donor-derived CAR-T cells were used in two independent experiments with at least 5 animals/treatment group.

### Transcriptome analysis

To quantify relative gene expression, the nCounter® CAR-T Characterization Panel (XT-CSO-CART1-12, Nanostring) kit was used. Following manufacturer’s instruction, we prepared cell lysate by re-suspending freshly sorted CAR-T cells at the same concentration (at least 5000 cells/µl) in modified RLT buffer (1/3 RLT buffer from Promega, 2/3 double deionized H_2_0). Hybridization, experimental run on the nCounter SPRINT and data collection were performed following manufacturer’s instructions. A total of 4–5 samples/condition were used. For the analysis, the raw data were uploaded on the nSolver 4.0 software. We performed the advanced analysis with generation of an nCounter Advanced Analysis Report following QC and normalization using the software internal controls. In the cell-type profiling section, genes previously shown to be characteristic of various cell populations are used to measure these populations' abundance. We specifically focused on the T cell compartment.

For the differential gene analysis focusing on migration, proliferation and cytotoxicity, the analyses were performed in the statistical R environment making use of the Bioconductor packages nanoStringNorm v1.2.1 for preprocessing and normalization, while the *limma* Bioconductor package was used for identifying differentially expressed genes. *P* values were calculated for adjusted values.

### Animal studies

6–8-week-old female NOD SCID Gamma (NSG) mice (Charles Rivers Laboratories) received 2 × 10^6^ MDA-MB-231 engineered to express firefly luciferase (ffLuc+) in PBS subcutaneously in the mammary fat pad (TNBC xenograft models). Luciferase expression was detected via intraperitoneal injection of 200 µg/mouse of D-Luciferin (Melford, L37060-1.0) and imaged using the IVIS Imaging System 100 Series (Perkin Elmer) at time points specified in figure legends. Living Image 4.4 software (Perkin Elmer) was used to quantify, normalize and compare bioluminescence imaging (BLI) signal. Mice received one single intravenous injection of 4 × 10^6^ CAR-T cells or equivalent non-transduced T cells. Refer to figure legends for exact time, dosage and additional therapeutics administered. Mice were carefully monitored and weighed every two days before and up to two weeks post-CAR-T cell treatment. Tumor measurements using a digital caliper started once tumors were visible by eye and continued up to the limit of the animal license (1.5 cm^3^) before mice were killed.

## Discussion

TNBC is an aggressive disease that is associated with a high incidence of metastasis and poor response to standard of care therapies. TNBC cells show high-level ROR1 expression, thus raising the potential for ROR1-targeting immunotherapies. A phase I study with ROR1 CAR-T cells demonstrated effective CAR-T cell infiltration into TNBC tumors but minimal efficacy due to upregulation of activation/exhaustion markers [11]. To overcome this limitation, we have designed an approach that overcome the immunosuppressive TME to enhance successful treatment of TNBC using CAR-T cells [12]. We engineered ROR1 CAR-T cells to allow rapid inducible secretion of PD-1-blocking scFv upon activation (F i-CAR-T cells). In vitro assays demonstrated enhanced cytotoxicity with this construct. Additionally, murine TNBC-like xenograft studies demonstrated that a single dose of F i-CAR-T slowed tumor growth and enhanced survival compared to control cohorts. Mechanistic studies showed increased numbers of tumor-infiltrating F i-CAR-T cells, with gene expression profiling showing upregulation of genes associated with migration, proliferation and cytotoxicity, suggesting that inducible PD-1 blockade improves F i-CAR-T cell fitness. Our study suggests that inducible expression of an anti-PD-1 scFv in the TME results in enhanced anti-tumor response when compared with conventional F CAR-T cells combined with systemic anti-PD-1 monoclonal antibody. There are a number of potential possibilities for why this may be the case, including localized delivery of PD-1 blockade within the direct tumor T cell junction. In addition, the use of an scFv versus a full-length antibody circumvents FcR-mediated degradation of CPB antibodies via tumor-associated macrophages within the TME [13], while also limiting the potential for dose-limiting toxicities associated with systemic administration.

A limitation of our in vivo studies is the use of a single preclinical TNBC model. Future studies will need to show efficacy of F i-CAR-T cells in a wider repertoire of ROR1 + cancer models. Nevertheless, our study provides the first proof-of-principal data supporting targeted delivery of a blocking anti-PD-1 scFv in TME leading to enhanced efficacy, thus addressing in part the limited efficacy of current CAR-T cell therapies in ROR1-positive malignancies, specifically TNBC tumors.

## Supplementary Information

**Additional file 1: Fig. S1**. Assessment of anti-PD-1 scFv binding. (A) Transgene schematic of bicistronic vector for constitutive secretion of anti-PD-1 scFv; (B) Supernatant from transfected HEK-293T cells producing anti-PD-1scFv was tested to confirm binding to PD-1+SupT1 cell line via flow cytometry; (C) Binding affinity measured via SPR using an anti-PD-1 scFv-Fc coated CM5 Chip: representative sensogram (of 3 independent repeats) of response units (RU) to increasing concentrations of PD-1 protein (0µg/ml–2.5µg/ml). **Fig. S2**. Transduction efficiency and additional in vitro characterization of F CAR and F i-CAR-T cells effector functions. (A) Representative flow cytometry plots of F CAR and F i-CAR T cells demonstrating comparable transduction levels between constructs as assessed by mCherry expression; (B) Representative flow cytometry of ROR1 and PD-L1 expression on positive MDA-MB-231, H1975 and negative control SUPT1 cell lines; (C) Anti-PD1 scFv was Luciferase-tagged and co-culture conditions were tested for scFv production via addition of substrate (coelenterazine) and luminescence quantified. Results are mean from 3 donors in triplicate; (D) Cytotoxicity assay following 72h of co-culture with CAR-T cells, viable ROR1- target cell lines were assessed via flow cytometry, results are mean+SD of 3 donors in triplicates normalized to control CD19 CAR-T cells; (E) Immune checkpoint expression was assessed via flow cytometry and shown as % of CAR-T cells. 2way ANOVA used: *p<0.05; **p<0.005. **Fig. S3**. In vivo monitoring of CAR-T cell treated mice. (A) BLI to assess tumors before and 4 weeks after treatment, 3-4 representative mice/group; (B) Tumor measurements (via caliper) up to day 37 post treatment, the arrow pointing at time point showed in Figure 2A, mean+SD of 6 mice/group; (C) Mice were monitored before and up to two weeks post CAR-T cell treatment for signs of CAR-T cell therapy-related side effects including weight loss (n=6/group shown); (D) MDA-MD-231 tumors were harvested at the end of the experiment and processed from tumor-bearing mice: immunohistochemistry analysis of tumors: a total of 5 tumors/group were stained for human CD8 and 5 sections/tumor were analyzed and quantified. One representative section for human CD8 from each treatment group. Positive control from human tonsil, negative control from untreated tumor. **Fig. S4.** Ex-vivo analysis shows that F i-CAR-T cells have a distinct transcription signature. (A) Heatmap showing raw numbers of T cell population frequencies (Log2 scale) from Nanostring analysis, including characteristic genes that were used to measure these populations' abundance (B) Pathway score comparisons between F CAR and F i-CAR T cells isolated from MDA-MB-231 tumors shown as box and whisker plots. Pathway scores are fit using the first principal component of each gene set’s data and oriented so that an increasing score corresponds to increasing expression (specifically, each pathway score has positive weights for at least half its genes). Data was analyzed through NanostringTM software.

## Data Availability

The data presented in this article are available upon request at the principal investigator’s laboratory.

## Abbreviations

CAR: Chimeric antigen receptor
CPB: Checkpoint blockade
CTLA-4: Cytotoxic T-lymphocyte antigen 4
ELISA: Enzyme-linked immunosorbent assay
FcR: Fc receptor
i-CAR: CAR-T cells secreting anti-PD-1 scFv
IFNγ: Interferon γ
IHC: Immunohistochemistry
IL-2: Interleukin 2
kDa: Kilo Daltons
LAG-3: Lymphocyte-activation gene 3
mAb: Monoclonal antibody
NFAT: Nuclear factor of activated T cells
NSCLC: Non-small cell lung cancer
NSG: Nod SCID gamma
PD-1: Programmed cell death receptor
PD-L1: Programmed cell death receptor-ligand 1
ROR1: Receptor tyrosine kinase-like orphan receptor 1
scFv: Single-chain variable fragment
SPR: Surface plasmon resonance
TILs: Tumor-infiltrating lymphocytes
TIM-3: T cell immunoglobulin and mucin-domain containing-3
TME: Tumor microenvironment
TNBC: Triple-negative breast cancer
