# Supplementary material for: Inducible localized delivery of an anti-PD-1 scFv enhances anti-tumor activity of ROR1 CAR-T cells in TNBC
Source: Breast Cancer Res. 2022 Jun 3;24:39. doi: 10.1186/s13058-022-01531-1 (PMC9166313; doi:10.1186/s13058-022-01531-1)
Supplement: Supplementary file 1 — Additional file 1: Fig. S1. Assessment of anti-PD-1 scFv binding. (A) Transgene schematic of bicistronic vector for constitutive secretion of anti-PD-1 scFv; (B) Supernatant from transfected HEK-293T cells producing anti-PD-1scFv was tested to confirm binding to PD-1+SupT1 cell line via flow cytometry; (C) Binding affinity measured via SPR using an anti-PD-1 scFv-Fc coated CM5 Chip: representative sensogram (of 3 independent repeats) of response units (RU) to increasing concentrations of PD-1 protein (0µg/ml–2.5µg/ml). Fig. S2. Transduction efficiency and additional in vitro characterization of F CAR and F i-CAR-T cells effector functions. (A) Representative flow cytometry plots of F CAR and F i-CAR T cells demonstrating comparable transduction levels between constructs as assessed by mCherry expression; (B) Representative flow cytometry of ROR1 and PD-L1 expression on positive MDA-MB-231, H1975 and negative control SUPT1 cell lines; (C) Anti-PD1 scFv was Luciferase-tagged and co-culture conditions were tested for scFv production via addition of substrate (coelenterazine) and luminescence quantified. Results are mean from 3 donors in triplicate; (D) Cytotoxicity assay following 72h of co-culture with CAR-T cells, viable ROR1- target cell lines were assessed via flow cytometry, results are mean+SD of 3 donors in triplicates normalized to control CD19 CAR-T cells; (E) Immune checkpoint expression was assessed via flow cytometry and shown as % of CAR-T cells. 2way ANOVA used: *p<0.05; **p<0.005. Fig. S3. In vivo monitoring of CAR-T cell treated mice. (A) BLI to assess tumors before and 4 weeks after treatment, 3-4 representative mice/group; (B) Tumor measurements (via caliper) up to day 37 post treatment, the arrow pointing at time point showed in Figure 2A, mean+SD of 6 mice/group; (C) Mice were monitored before and up to two weeks post CAR-T cell treatment for signs of CAR-T cell therapy-related side effects including weight loss (n=6/group shown); (D) MDA-MD-231 tum [file 13058_2022_1531_MOESM1_ESM.pdf]

Figure S1

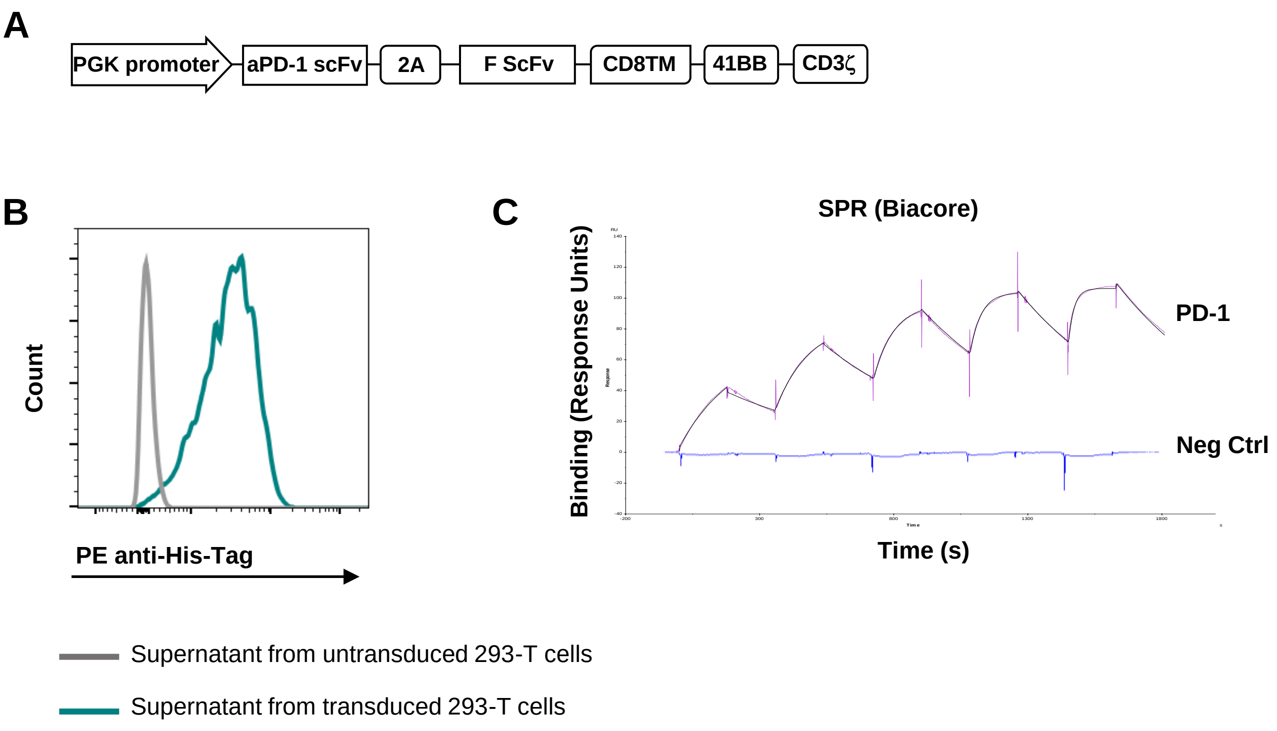
A
PGK promoter
aPD-1 scFv
2A
F ScFv
CD8TM
41BB
CD3ζ
B
Count
PE anti-His-Tag
C
SPR (Biacore)
Binding (Response Units)
PD-1
Neg Ctrl
Time (s)
Supernatant from untransduced 293-T cells
Supernatant from transduced 293-T cells

Figure S2

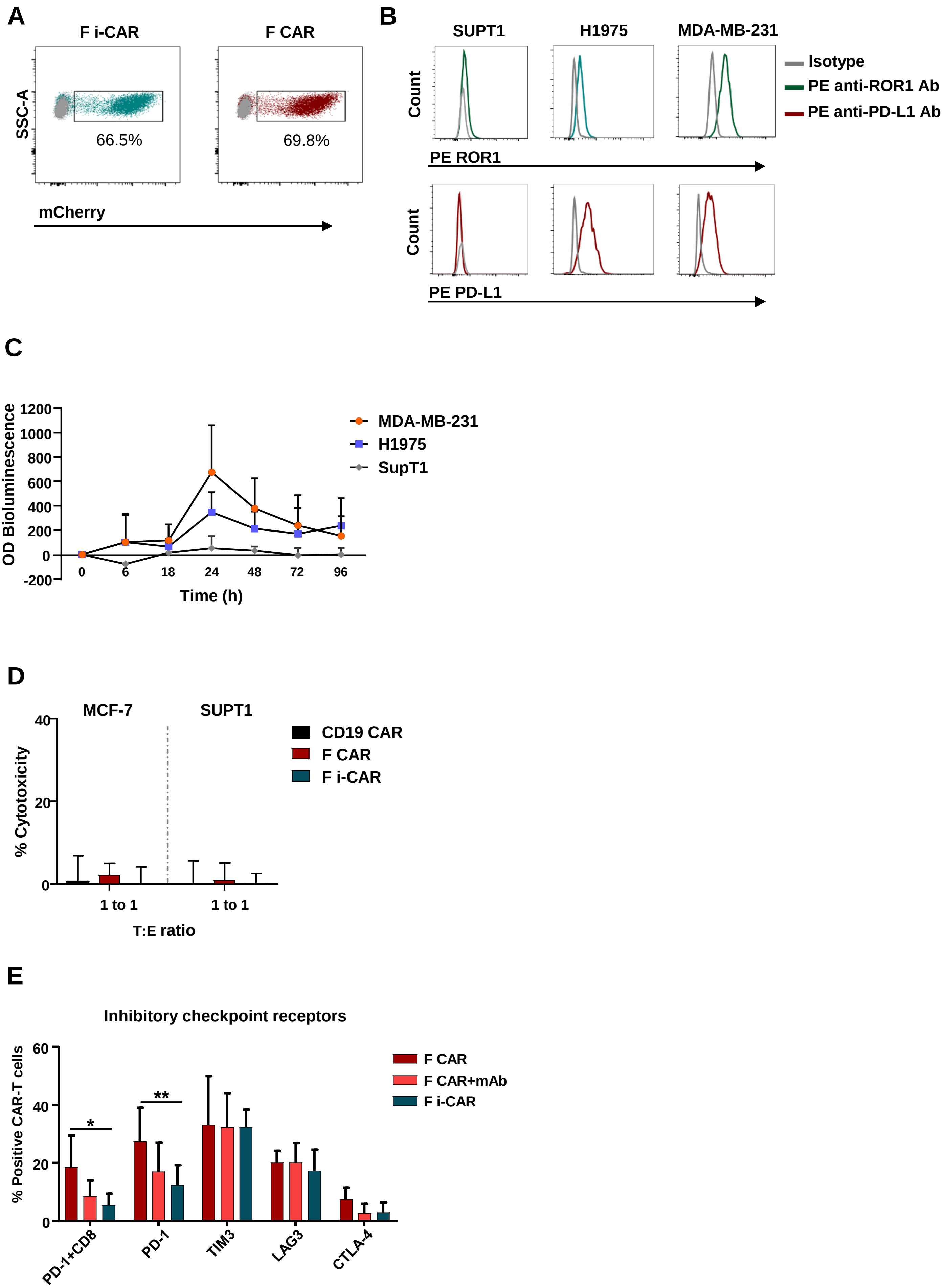

A
F i-CAR
F CAR
SSC-A
66.5%
69.8%
mCherry
B
SUPT1
H1975
MDA-MB-231
Count
Isotype
PE anti-ROR1 Ab
PE anti-PD-L1 Ab
PE ROR1
PE PD-L1
C
OD Bioluminescence
1200
1000
800
600
400
200
0
-200
0
6
18
24
48
72
96
Time (h)
MDA-MB-231
H1975
SupT1
D
MCF-7
SUPT1
40
20
0
% Cytotoxicity
1 to 1
1 to 1
T:E ratio
CD19 CAR
F CAR
F i-CAR
E
Inhibitory checkpoint receptors
% Positive CAR-T cells
60
40
20
0
*
**
PD-1+CD8
PD-1
TIM3
LAG3
CTLA-4
F CAR
F CAR+mAb
F i-CAR

Figure S3

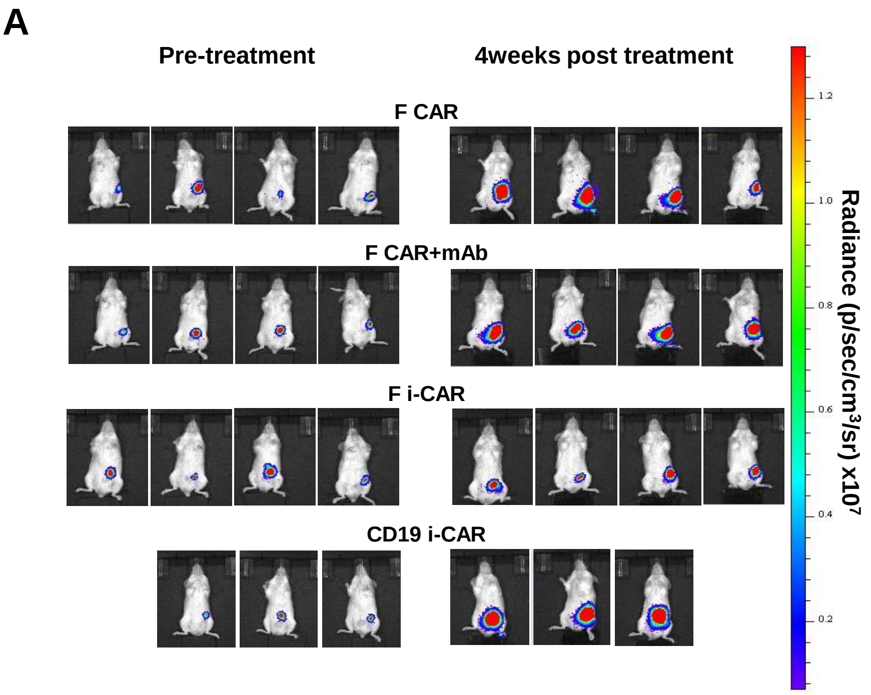

A
Pre-treatment
4weeks post treatment
F CAR
F CAR+mAb
F i-CAR
CD19 i-CAR
Radiance (p/sec/cm³/sr) x10⁷
1.2
1.0
0.8
0.6
0.4
0.2

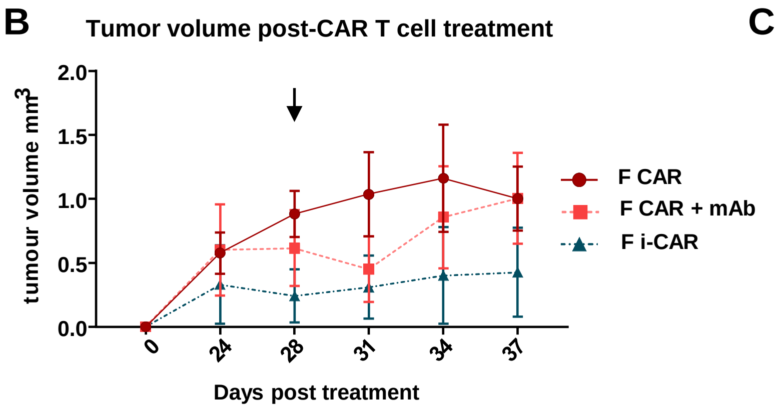

B
Tumor volume post-CAR T cell treatment
tumour volume mm³
2.0
1.5
1.0
0.5
0.0
0
24
28
31
34
37
Days post treatment
F CAR
F CAR + mAb
F i-CAR

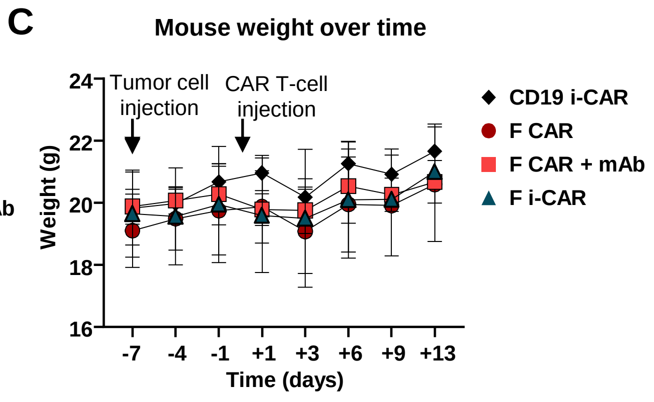

C
Mouse weight over time
Tumor cell injection
CAR T-cell injection
Weight (g)
24
22
20
18
16
-7
-4
-1
+1
+3
+6
+9
+13
Time (days)
CD19 i-CAR
F CAR
F CAR + mAb
F i-CAR

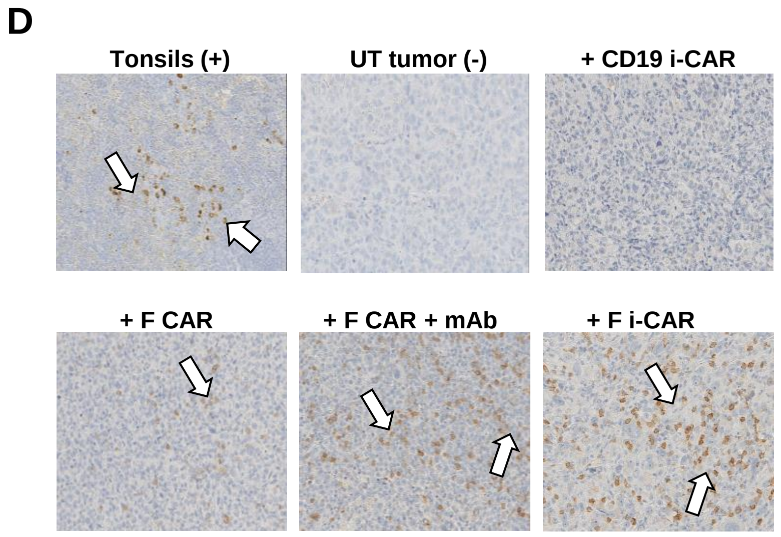

D
Tonsils (+)
UT tumor (-)
+ CD19 i-CAR
+ F CAR
+ F CAR + mAb
+ F i-CAR

Figure S4

A

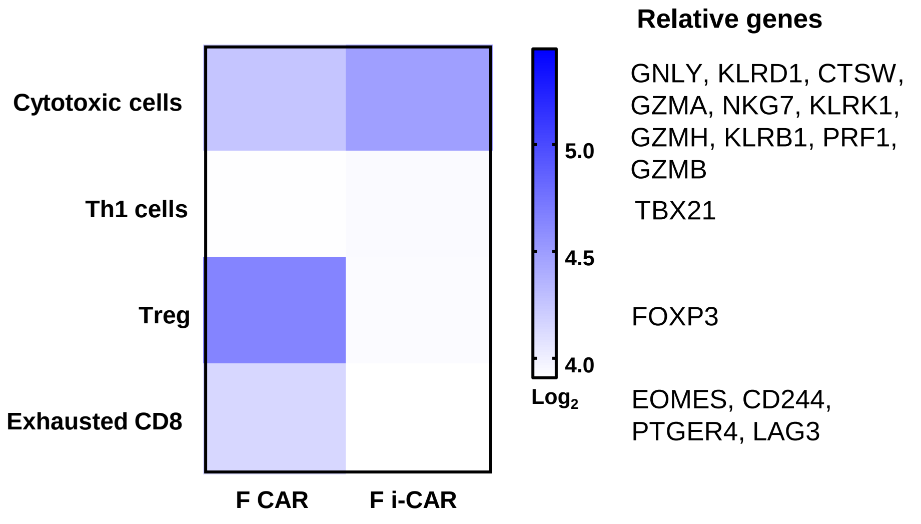
Relative genes
Cytotoxic cells
Th1 cells
Treg
Exhausted CD8
F CAR
F i-CAR
5.0
4.5
4.0
Log2
GNLY, KLRD1, CTSW, GZMA, NKG7, KLRK1, GZMH, KLRB1, PRF1, GZMB
TBX21
FOXP3
EOMES, CD244, PTGER4, LAG3

B

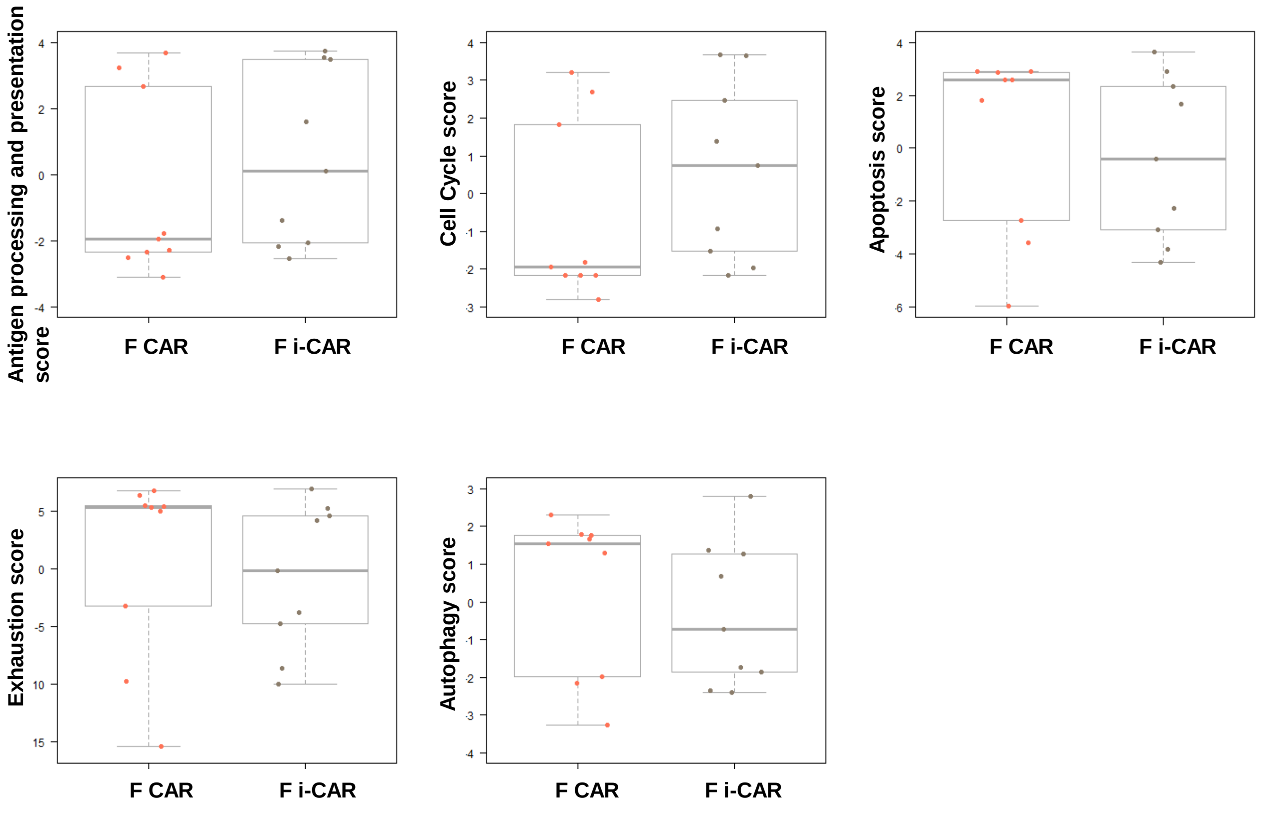
Antigen processing and presentation score
Cell Cycle score
Apoptosis score
Exhaustion score
Autophagy score
F CAR
F i-CAR
